# Human exceptional longevity: transcriptome from centenarians is distinct from septuagenarians and reveals a role of Bcl-xL in successful aging

**DOI:** 10.18632/aging.101078

**Published:** 2016-10-28

**Authors:** Consuelo Borras, Kheira M. Abdelaziz, Juan Gambini, Eva Serna, Marta Inglés, Monica de la Fuente, Idoia Garcia, Ander Matheu, Paula Sanchís, Angel Belenguer, Alessandra Errigo, Juan- Antonio Avellana, Ana Barettino, Carla Lloret-Fernández, Nuria Flames, Gianni Pes, Leocadio Rodriguez-Mañas, Jose Viña

**Affiliations:** ^1^ Facultad de Medicina, Universidad de Valencia, Valencia, Spain, INCLIVA and Spanish Centenarian Study Group; ^2^ Facultad de Fisioterapia Universidad de Valencia, Valencia, Spain; ^3^ Facultad de Ciencias Biológicas, Universidad Complutense de Madrid, Madrid, España; ^4^ Instituto Biodonostia, San Sebastian, Spain; ^5^ IIKERBASQUE, Basque Foundation for Science, Bilbao, Spain; ^6^ Servicio de Geriatría. Hospital de la Ribera. Alzira, Valencia, Spain; ^7^ Dipartimento di Medicina Clinica e Sperimentale, Viale San Pietro 8, I-07100 Sassari, Italy; ^8^ Instituto de Biomedicina de Valencia, IBV-CSIC, 46010 Valencia, Spain; ^9^ Departamento de Geriatría. Hospital Universitario de Getafe, Madrid, Spain

**Keywords:** healthy aging, apoptosis, Bcl-2, FAS ligand, longevity, RNA

## Abstract

Centenarians not only enjoy an extraordinary aging, but also show a compression of morbidity. Using functional transcriptomic analysis of peripheral blood mononuclear cells (PMBC) we identified 1721 mRNAs differentially expressed by centenarians when compared with septuagenarians and young people. Sub-network analysis led us to identify Bcl - xL as an important gene up-regulated in centenarians. It is involved in the control of apoptosis, cellular damage protection and also in modulation of immune response, all associated to healthy aging. Indeed, centenarians display lower plasma cytochrome C levels, higher mitochondrial membrane potential and also less cellular damage accumulation than septuagenarians. Leukocyte chemotaxis and NK cell activity are significantly impaired in septuagenarians compared with young people whereas centenarians maintain them. To further ascertain the functional role of Bcl- xL in cellular aging, we found that lymphocytes from septuagenarians transduced with Bcl-xL display a reduction in senescent-related markers. Finally, to demonstrate the role of BcL-xL in longevity at the organism level, *C. elegans* bearing a gain of function mutation in the BcL-xL ortholog *ced-9,* showed a significant increase in mean and maximal life span. These results show that mRNA expression in centenarians is unique and reveals that BcL- xL plays an important role in exceptional aging.

## INTRODUCTION

The rapidly graying of populations has stimulated governments all over the world to switch the focus of health care, from delivering adequate treatments to patients with acute episodes of single diseases to providing good health to individuals faced with multiple chronic conditions [1]. Some elderly people themselves may provide clues toward how to achieve healthy aging. Centenarians, for example, exhibit medical histories with remarkably low incidence rates of common age-related disorders such as vascular-related diseases, diabetes mellitus, Parkinson's disease, and cancer [2]. Over 80% of centenarians delay their first experience of diseases often associated with high mortality till beyond the age of 90 years or escape these morbidities entirely [2]. Moreover, centenarians may have better cognitive function and require minimal assistance for activities of daily living compared with younger elderly who exhibit normal aging [3]. Thus in individuals who reach the maximum limit of human life span, morbidity is compressed toward the end of life (that is, health span approximates life span) and then there is a rapid onset of decline in functional status and organ reserve; centenarians are an example of successful aging [3].

The Spanish Centenarian Study Group, founded in 2007 as a population-based research program focused on centenarians living in various areas of Spain, including that of La Ribera near Valencia, Spain, previously investigated molecular mechanisms by which centenarians maintain homeostasis and thereby evade age-related morbidities as evidenced by changes in their microRNA (miRNA) expression profiles in peripheral blood mononuclear cells (PBMCs) [4]. PBMCs are the most easily accessible human tissue and the only cells ethically and noninvasively available from very elderly subjects in sufficient amounts to obtain workable yields of RNA. Moreover, PBMCs are a useful cell model for genomics studies with over 80% co-expression of genes similarly up-regulated in other human target tissues in response to environmental stimuli [5]. PBMCs have thus been described as “sentinel” biomarkers ideal as surrogates for organism-wide genetic regulation [5].

Our previous analysis of miRNA microarray data (“miRNome”) showed that miRNA expression in centenarians (successful aging) exhibited significant overlap with that in young people but not with septuagenarians (normal aging) [4]. We observed that centenarians overexpress seven small noncoding RNAs of which four (scaRNA-17, mir-21, mir-130a, and mir-494) are known to be associated with a range of health-beneficial and life span-enhancing actions including telomerase over-expression in Cajal bodies, neuro-protection in ischemia, cardioprotection, and inhibition of mitochondrial damage and apoptosis [6-9].

We thus hypothesized that expression patterns of mRNAs in centenarians versus septuagenarians and young people might provide further insights into what discriminates those with exceptional longevity from normal aging. In the present study we sought to identify expression patterns of mRNAs in centenarians as means to elucidate factors that influence why these individuals live such long, healthy lives. We have identified Bcl-xL as one of these factors that influence longevity in humans.

## RESULTS

### mRNA expression in human exceptional longevity significantly differs from that of ordinary aging

The present investigation began by looking at how mRNA expression is controlled in centenarians. We performed functional transcriptomic analysis using Genechip Human Gene 1.0ST array to analyze expression patterns of 28,869 human genes. As with the miRNA data [4], we found that the mRNome of centenarians and septuagenarians is very different, with over 12,000 mRNAs expressed differentially (at a level of significance P<0.05 using false discovery rate—a statistical test designed specifically to analyze micro-array data) between these two populations (Supplementary Fig. 1a).

**Figure 1:**
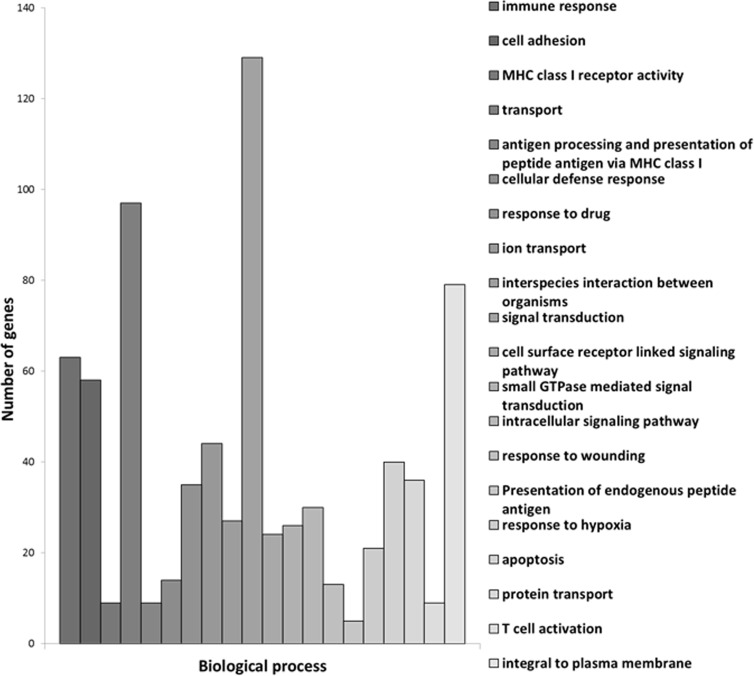
Centenarians characteristic genes grouped in terms of biological processes In the X axis we show the biological processes implicated and in the Y axis the number of genes involved.

Furthermore, we identified 1721 genes that were characteristic of centenarians because they were differentially expressed when compared with septuagenarians and young people (Supplementary Fig. 1b). We have grouped those genes in terms of biological processes. These are shown in Figure 1. In this Figure, in the Y axis we show the number of genes involved and in the X axis the biological processes implicated. We have grouped these processes in terms of a p value with the highest p values shown on the left side of the figure. Therefore, the highest p value that we observed is the immune response followed by cell adhesion, and major histocompatibility complex class 1 receptor activity, transport processes, etc.

These groupings give an idea of the major processes that may be involved in exceptional longevity. Of course, our further analysis of sub-networks has highlighted the role of genes involved in apoptosis, a process that may be relevant in several others, like immune response. The list of the hundred processes that are preferentially involved in exceptional longevity (the first twenty of which are shown in Figure 1) is shown in Supplementary Table 1.

Among the twenty processes where we find a higher significance of the centenarians’ gene expression versus septuagenarians or young persons, two major processes emerge. The first is immune response and the second is signalling. For instance, immune response is involved as a process itself but also because major histo-compatibility complex receptor is a major process that appears in the list as well as cell adhesion, antigen processing, presentation of endogenous peptide antigens, and T cell activation. This is in keeping with the immune response theory of ageing [10]. Moreover, our further experiments (see Figure 4) indicate that centenarians have an extraordinary immune response that make them similar to young persons and different from septuagenarians.

Cell signalling is another group of processes that emerges as important in extraordinary aging (centenarians). The conclusion that can be drawn is that centenarians maintain a more accurate cell signalling network than septuagenarians. We have previously proposed the cell signalling disruption theory of ageing [11]. The results reported in this paper are thus in keeping with the idea that the cell signalling network is deranged in ordinary ageing and maintained in an extraordinary one. Moreover, we have previously described [4] that the microRNAome of centenarians is similar to young ones and very different from octogenarians. Because micro-RNAs are involved in cell signalling and in the control of mRNA expression, the fact that cell signalling is an important process that is maintained in extraordinary ageing, may be, at least in part, explained because centenarians maintain a high expression of microRNAs, one that is lost in septuagenarians.

**Figure 2:**
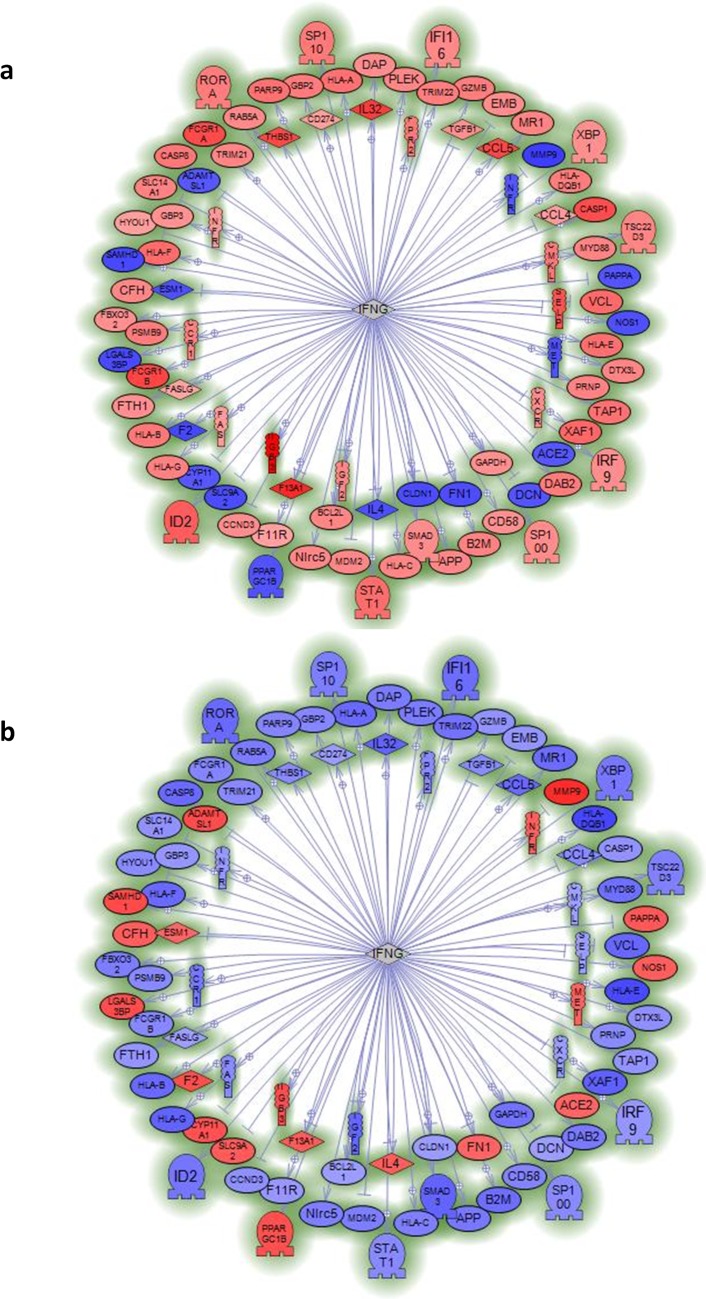
Sub-network analysis of genes specifically regulated by centenarians or septuagenarians versus young subjects indicates the relevance of interferon gamma (IFNG) Sub-network of genes regulated by interferon gamma (IFNG) in mononuclear cells from centenarians (**a**) or septuagenarians (**b**) versus young subjects. Data reported in Supplementary Figures 2–7 show other sub-network analyses that point to TNF, TCR, SP1, TGFB1, and IL-32 as genes with regulatory importance that are specific for centenarians. Sub-networks are generated by connecting genes to their neighbors in the database (ResNet 8.0; 2010Q4 Mammal). Sub-networks with the best P-value of 0.05 enrichment P-value cutoff were selected.

We also employed Ariadne software package (since acquired by Elsevier B.V. and renamed Pathway Studio) to perform a sub-network analysis on these 1721 mRNAs in order to group them. A sub-network is a group of genes structurally or functionally connected to a common gene; a sub-network is identified when there are known relations among genes recorded on the Ariadne database. Our sub-network analysis converged on six genes: interferon (IFN)-γ (IFNG) ; T-cell receptor (TCR); tumor necrosis factor (TNF); SP1 transcription factor (SP1); transforming growth factor (TGF)-β1 (TGFβ1); and a cytokine, namely, IL-32 (Fig.2 and Supplementary Fig. 2-7). When we compared centenarians with young people and septuagenarians with young people, we observed an almost mirror image: genes that were up-regulated in centenarians tended to be down-regulated in septuagenarians thus suggesting that activation of these networks are associated with exceptional aging. We conclude that mRNA expression in human exceptional longevity significantly differs from that of ordinary aging.

The genetic characteristics of extreme longevity have been thoroughly studied by the analysis of genetic variants (SNPs) [12], and more recently by epigenetic analysis [13], but to our knowledge no studies have been performed analysing global gene expression associated to extreme longevity in humans. Sarup et al. showed that flies selected for longevity retain a “young” gene expression profile. These results, in invertebrates, are in keeping with ours in human, i.e. that individuals displaying exceptional longevity up- regulate genes that are down regulated in ordinary aging [14].

### Analysis of the genes over-expressed in centenarians reveals a pattern of apoptotic- related genes

Analysis of the genes over-expressed in centenarians reveals a pattern of Using Ariadne software, we observed that the six genes identified in the sub-network analysis (interferon (IFN)-γ (IFNG) ; T-cell receptor (TCR); tumor necrosis factor (TNF); SP1 transcription factor (SP1); transforming growth factor (TGF)-β1 (TGFβ1); and a cytokine, namely, IL-32 ) were related to three genes: Bcl-xL (also known as BCL2L1), Fas and Fas ligand (FasL), all of them involved in the control of apoptosis. Moreover, using Gene Ontology we detected that apoptosis is one of the processes most commonly conserved in centenarians. Fas and FasL are mainly involved in the control of the extrinsic pathway to apoptosis, whereas Bcl-xL inhibits the intrinsic, mitochondrial pathway to apoptosis [15]. Binding of Fas ligand to Fas receptor activates the caspase enzymatic cascade resulting in cleavage of a variety of target proteins with structural or regulatory functions and destruction of the cell [16]. Alternately, in the int-rinsic apoptotic pathway different signals converge on mitochondria stimulating these organelles' release of caspase activators such as cytochrome c. Intrinsic apoptosis is believed to occur in response to a variety of perturbations of intracellular homeostasis including DNA damage and oxidative stress, processes well known to accumulate with aging[17, 18]; Bcl-xl belongs to the big family of BCL-2 proteins. They are characterized by the presence of BCL-2 Homology (BH) domains, and are grouped into three subfamilies based on the number of BH domains they share. The first subfamily includes the anti-apoptotic proteins BCL-2, BCL-xL, BCL-w, MCL-1 and A1/BFL-1. These proteins possess four BH domains—BH1–4. Other subfamilies are pro-apoptotic proteins. Those which possess three BH (BH1–3) domains are represented by BAX, BAK and BOK and those characterized by the presence of only the BH3 domain. Therefore, BCL-2 family proteins form a complex regulatory network that controls cell survival and death in response to different physiological and pathological stimuli [19].

Bcl-xL down regulates apoptosis and promotes cell survival by migrating to mitochondrial outer membrane, counteracting mitochondrial permeabilization (pore formation) activity by BAX and BAK, and inhibiting cytotoxic adaptors needed for activation of caspases that dismantle the cell [20, 21]. Indeed, Bruey et al showed that Bcl-xL binds and suppress NALP1, reducing caspase-1 activation and interleukin-1b (IL-1b) production [22].

### Parameters associated to Bcl-xL and healthy aging are preserved in centenarians

Bcl-xL is involved not only in the control of apoptosis, but also in mitochondrial damage protection [23], modulation of immune response [24], control of mitochondrial respiration [25]and DNA repair [26] All of these processes are associated to healthy aging [27].

**Figure 3:**
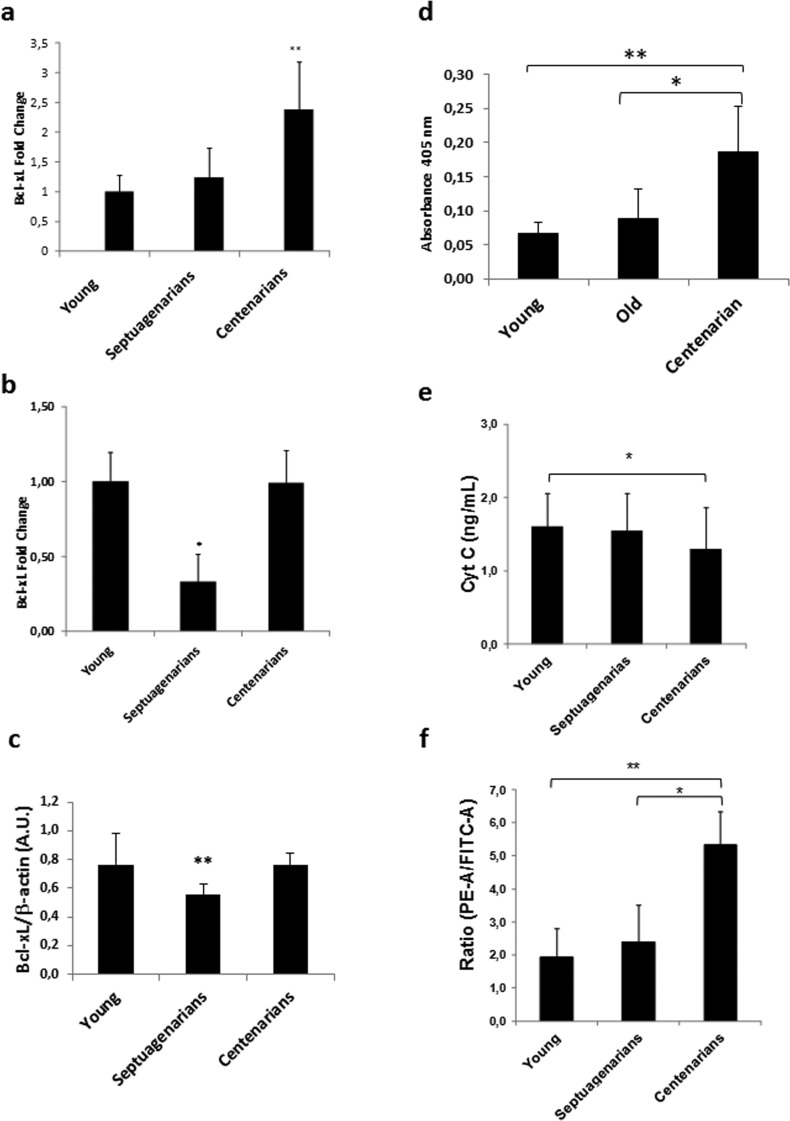
Peripheral blood mononuclear cells (PBMCs) from centenarians over-express Bcl-xL and exhibit decreased markers of intrinsic apoptosis (**a**) Bcl-xL expression in PBMCs of Alzira centenarians. RT-PCR analysis in peripheral blood mononuclear cells from young (n=14), septuagenarians (n=6), or centenarians (n=19) reveals over-expression of Bcl-xL in centenarians

We evaluated centenarians' expression of BcL-xL by RT-PCR and confirmed that it is indeed up-regulated compared with septuagenarians and young people (Fig. 3a).

To validate the results obtained in the Spanish cohort, we measured BcL-xL expression in another well characterized centenarian population, i.e., that of the Sardinian centenarians [28]. We found that, as in the Spanish cohort, Sardinian centenarians display a higher Bcl-xL expression than septuagenarians and maintain an expression similar to young individuals (Fig. 3b). The same pattern is shown when measuring Bcl-xL protein expression (Fig. 3c).

Since our gene microarray analysis indicated that the extrinsic pathway of apoptosis may be highly activated in centenarians as evidenced by their up-regulated levels of Fas and FasL mRNAs, we further ascertained that caspase 8 activity was also higher in centenarians than septuagenarians and young people (Figure 3d). However, we did not find differences in caspase 3 and 9 (results not shown) suggesting that extreme longevity in centenarians does not affect the final common pathway of apoptosis. The general picture that emerges from our series of experiments is that centenarians have an intact extrinsic pathway of apoptosis thus killing cells that may be damaged by environmental insults but down-regulated intrinsic apoptosis thus sparing cells that have not been exposed to genotoxic or other challenges. Upregulation of Bcl-xL as noted in our gene expression studies suggests that regulation of apoptosis is deranged in septuagenarians (normal aging) yet preserved in centenarians (exceptional aging). Thus, to further characterize apoptosis in centenarians we obtained blood from our cohort of subjects and observed that plasma cytochrome c, a systemic marker of intrinsic apoptosis [29], was maintained at low levels in centenarians but not in septuagenarians (Fig. 3e). Moreover, mitochondrial membrane potential (ΔΨm) as assessed by JC-1 cytometry [30] was significantly higher in PBMCs obtained from centenarians versus septuagenarians as well as young people (Fig. 3f), suggesting that the functional state of mitochondria was maintained and hence intrinsic apoptosis minimized in centenarians.

**Figure 4:**
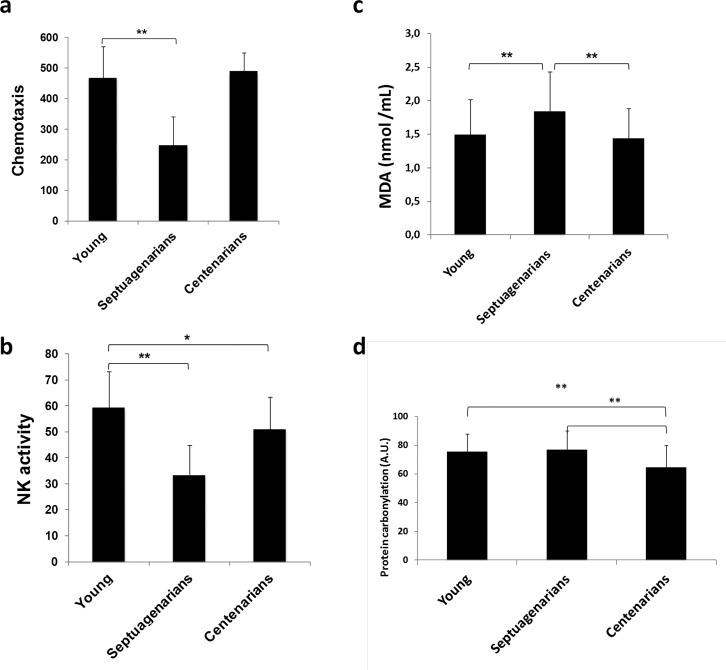
Lymphocyte function is preserved in centenarians (**a**) Lymphocyte chemotaxis and (**b**) Natural killer (NK) cell activity assessed in blood samples obtained from young (n=70), septuagenarians (n=70), and centenarians (n=70). (**c**) Lipid peroxidation marker malondialdehyde (MDA) levels in plasma derived from young subjects (n=31), septuagenarians (n=31), and centenarians (n=27) as determined by HPLC. (**d**) Protein carbonylation levels in plasma derived from young subjects (n=31), septuagenarians (n=31), and centenarians (n=27) as determined by western blotting. Data are expressed as mean ± SD. *P<0.05; **P<0.01.

As stated before, BcL- xL is important in the development and maintenance of the immune system [24]. Moreover, immunosenescence (age-related decline of immune function) has been posited responsible at least in part for the well-known increased incidence rates of infections, cancer, and autoimmune diseases that arise in elderly persons who display normal aging [31] [32]. We thus analyzed lymphocyte function in centenarians and showed that leukocyte chemotaxis and NK cell activity were significantly impaired in septuagenarians compared with young people whereas in centenarians these indicators of immunosenescence were similar to the picture noted in young people (Figs. 4a and 4b). Therefore, using centenarian-donated PBMCs, we observed a number of similarities between centenarians and young persons, which were not reflected in cells donated by septuagenarians, in a variety of biological factors suggestive that centenarians may evade the relentless onset of immunosenescence that is seen in normal aging. This is in keeping with the idea of immuneaging first postulated by Franceschi [33].

BcL-xL is a mitochondrial protein involved in the control of respiratory chain and thus in the rate of mitochondrial free radical production [34, 35]. We determined lipid peroxidation levels measured as malondialdehyde (MDA) and protein carbonylation and found that, in septuangenarians they are higher than in centenarians (Figs. 4c and 4d). This fits with the idea that oxidative damage is associated with loss of function in normal aging [11].

Taken together, our results demonstrate that, similar to what we found in microRNA expression, septuagenarians (normal aging) display a cell health impairment which is not so evident in young people or centenarians (exceptional aging). Moreover, they suggest that Bcl-xL may play a major role in healthy aging.

### Overexpresion of Bcl-xL decreases premature senescence in mouse embryo fibroblasts and in human isolated lymphocytes

**Figure 5:**
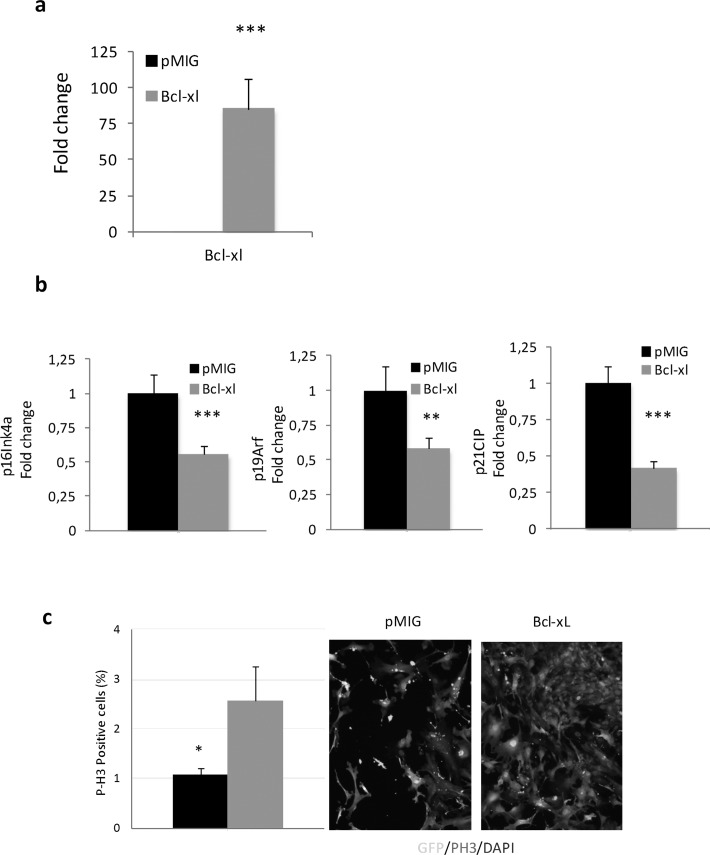

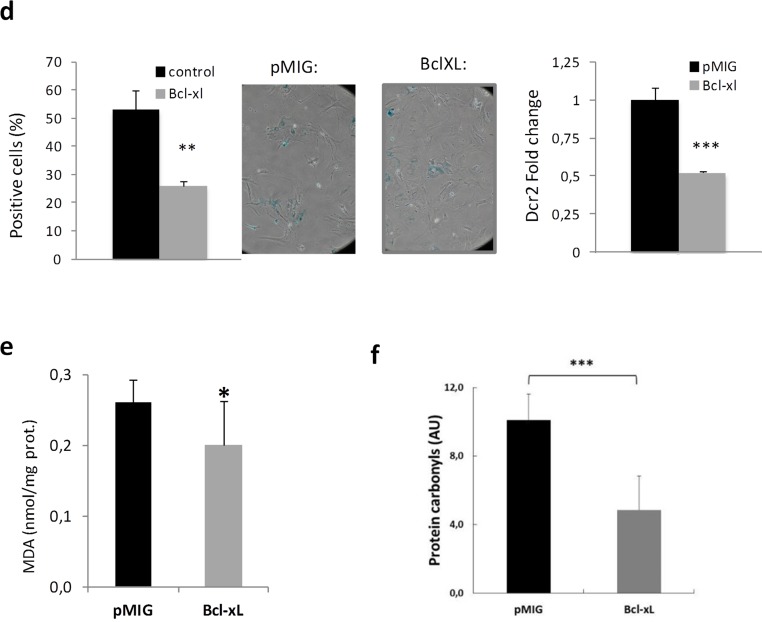
Bcl-xL, decreases senescence, enhances proliferation and protects against oxidative damage in mouse embryo fibroblasts (MEFs) in primary culture (**a**) Bcl-xLover-expression in MEFs. (**b**) Bcl-xLdown-regulates expression of age-associated cell cycle inhibitors: RT-PCR mRNA expression analysis of p16Ink4a, p19Arf, and p21CIP in MEFs retrovirally transduced withBcl-xLorpMIG (controls) (n=7 in each group). (**c**) Bcl-xL over-expression increase proliferation in MEFs (determined as P-H3 positive cells). (**d**) Bcl-xLover-expression prevents cellular commitment to senescence by decreasing SA-β-Gal and expression of Dcr2. SA-β-Gal activity was measured by SA-β-Gal staining kit (n=3 in each group). RT-PCR expression analysis of Dcr2 in MEFs virally transduced with Bcl-xL orpMIG (controls) (n=7 each). (**e**) MEFs transduced with Bcl-xL show lower levels of lipid peroxidation, determined as malondialdehyde, MDA by HPLC (pMIGcontrols, n=6; Bcl-xL, n=11) (**f**) MEFs transduced with Bcl-xL show lower levels of oxidized proteins as measured by western blotting (pMIGcontrols, n=6; Bcl-xL, n=11). Data are expressed as mean ± SD. *P<0.05; **P<0.01; ***P<0.001.

Cells in culture carry out a limited number of divisions before undergoing senescence, a process associated with mammalian aging [36]. In an effort to determine the functional role of BcL-xL on cellular senescence, we retrovirally transduced mouse embryo fibroblasts (MEFs) with a plasmid encoding BcL-xL in parallel with empty controls (pMIG). Interestingly, we did not observe any difference between them at early passage (data not shown), but after four passages in culture, BcL-xL overexpressing cells (Fig. 5a) exhibited lower accumulation of p16Ink4a, p14Arf, and p21Cip, all of them cell cycle inhibitors, see Fig. 5b and increased proliferation (Fig. 5c) compared to control cells. Moreover, ectopic expression of Bcl-xL significantly delayed the accumulation of senescent cells in the culture measured by a decrease in the number of senescence-associated β-galactosidase (SA-β-GAL) positive cells and lower levels of Dcr2 (another marker of senescence, Fig. 4d). Moreover, we observed that Bcl-xL–overexpressing MEFs were protected against oxidative damage as evidenced by low protein carbonylation and lipid peroxidation (measured as MDA), i.e. the same picture as that observed in centenarians (Fig. 5e and 5f). These data suggest that Bcl-xL confers protection against cellular damage accumulation.

**Figure 6:**
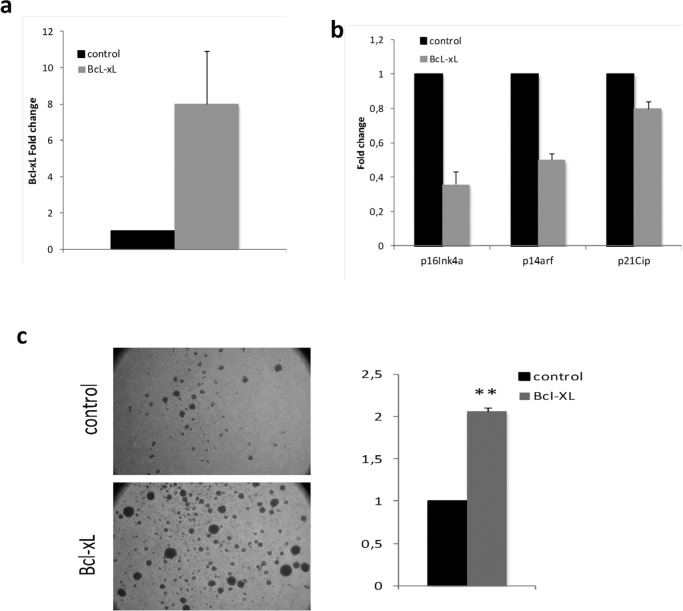
Bcl-xL, decreases senescence and enhances proliferation in lymphocytes from septuagenarian individuals in primary culture (**a**) Bcl-xL over-expression in lymphocytes from septuagenarian individuals. (**b**) Bcl-xL down-regulates the expression of age-associated cell cycle inhibitors p16Ink4a, p14Arf, and p21CIP in septuagenarian PBMCs transduced with Bcl-xL (n=3). (**c**) Representative pictures (left) and quantification of sphere formation (right) of PHA-stimulated lymphocytes infected with Bcl-xL or the empty vector (n=2).

In order to extend this analysis to a more relevant cellular setting, we transduced lymphocytes from septuagenarians with BcL-xL (Fig. 6a). We observed that p16Ink4a, p14Arfand p21Cipcell cycle inhibitors expression was lower in septuagenarian cells overexpressing BcL-xL than in controls (Fig. 6b). This correlates with their increased proliferation capacity stimulated by phytohaemaglutinin (PHA) (Fig. 6c). In these respect, cells from septuagenarians, when they overexpress Bcl-xL, behave like those from centenarians. Together, our data indicate that high levels of BcL-xL exert a beneficial effect in cellular aging and support the notion that BcL-xL could contribute to the exceptional longevity and healthspan of centenarians.

### Ced-9 (ortholog of human Bcl-xL) overexpression in *C. elegans* increases mean and maximum survival time

To assess if increased activity of Bcl-xL promotes longevity *in vivo* we turned to the simple model organism *C. elegans*. This nematode has several advantages for aging studies: it has a short life span of around twenty days, it shares the main hallmarks of human aging and around 70% of the human genome has a *C. elegans* ortholog, including the apoptotic pathway that was originally described in this organism [37, 38]).

**Figure 7:**
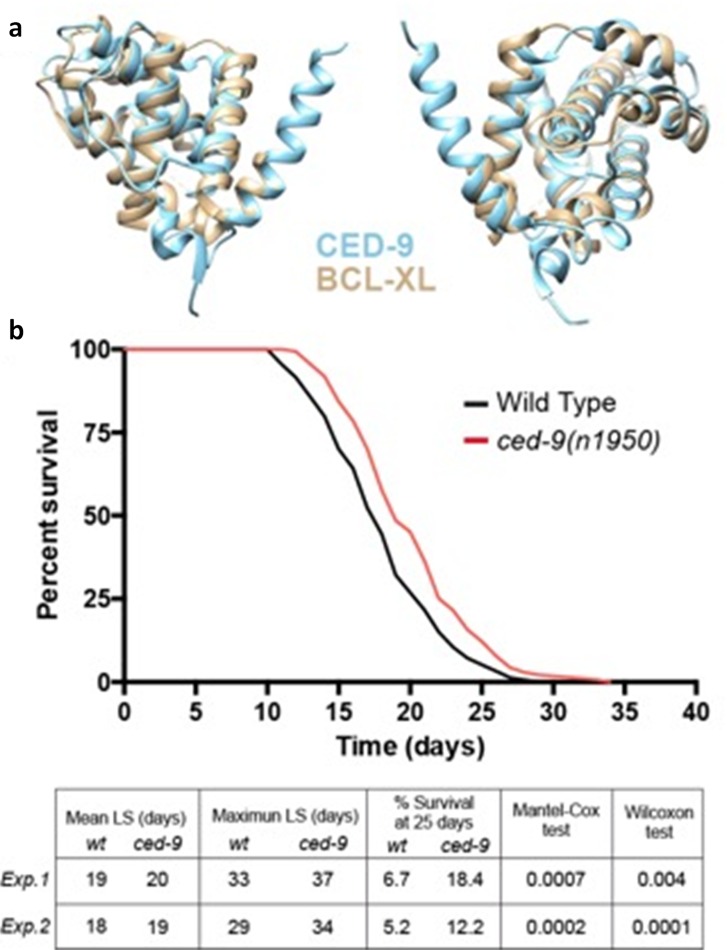
*C. elegans* baring a gain of function mutation in the Bcl-xLorthologced-*9(n1959)* show an increased longevity (**a**) *ced-9* and *Bcl-XL* protein domains by BCL-XL 3D modeling. (**b**) Representative wildtype and *ced-9(n1959)* gain of function allele longevity curve and Table with the data for two replicates *(Exp.1*: experiment 1; *Exp.2*: experiment 2). ced-9(n1950) animals show increased mean and maximum lifespan (LS) and double the percentage of alive animals at 25 days compared to wildtype. Statistical test refers to mean LS.

*ced-9* is the only *C. elegans* member of the Bcl2 anti-apoptotic family and thus the ortholog of human Bcl-xL, showing 44% homology and the same protein domains (Fig. 7a). However, it is important to state that there are differences between CED-9 and mammalian anti-apoptotic Bcl-2 homologs in terms of the dynamic behavior of BH3-binding hydrophobic cleft and the region that participates in the CED-4 binding activity. This should be taken into account in drug screening studies in which model organisms such as *C. elegans* are used [39].

Among the multiple available *ced-9* alleles, *ced-9(n1950)* is a missense G to A substitution that confers constitutive activity to the CED-9 protein [40]. We hypothesized that this mutation could mimic the increased Bcl-xL levels of centenarians and thus we performed longevity curves of *ced-9(n1950)* compared to wildtype worms. Interestingly, *ced-9(n1950)* animals showed a significant increase both in the mean and maximum survival time. Moreover, at 25 days, which can be considered a very advanced age for a worm, the percentage of *ced-9(n1050)* survivals was more than double compared to wildtype (Fig. 7b). This effect was already shown by Yee et al., although this finding was not highlighted by these authors as it was not the main purpose of their study [41].

## CONCLUSION

Our results demonstrate the functional relevance of increased levels of Bcl-xL at cellular and organismal level and support the notion that the role of Bcl-xL in exceptional aging is maintained across the evolution. However, as stated by Chondrogianni *et al*, [42] it remains very unlikely that a single gene (or even a gene family) is a universal biomarker of longevity. It is our opinion that longevity is an extremely multifactorial issue. Many genes may contribute to successful aging and longevity by providing cell survival and/or cell adaptation signals.

In sum, our findings provide an exciting glimpse as to how the very oldest persons in society achieve not only long lives but also healthy long lives. Our data on the centenarian mRNome (together with our previous findings on the centenarian miRNome [4]) imply that these oldest individuals seem to retain the ability to regulate genes that have been demonstrated involved in cellular survival, and identify Bcl-xL as a player in the protection against age-associated damage.

## METHODS

### Ethics statement

Investigation has been conducted in accordance with the ethical standards and according to the Declaration of Helsinki and according to the national and international guidelines and has been approved by the author's institutional review board.

### Study population

The Spanish Centenarian Study Group at RETICEF began in 2007 as a population-based study of all centenarians living within an area near of Valencia called La Ribera (11th Health Department of the Valencian Community, Spain), which comprises 29 towns (240,000 inhabitants). Potential subjects were selected from the population data system of the 11th Health Department. We found 31 centenarians of whom 20 met the inclusion criteria. Then, we randomly recruited 20 septuagenarians of whom 16 met the inclusion criteria and 20 young people of whom 14 fulfilled the inclusion criteria. The inclusion criteria were: to be born within the dates indicated in the study (before 1918 for centenarians, between 1928 and 1938 for septuagenarians, and between 1968 and 1988 for young individuals); to live in the 11th Health Department for the past ≥6 years; and to provide informed consent. The sole exclusion criterion was to be terminally ill for any reason.

All experimental procedures were approved by the Committee for Ethics in Clinical Research of the Hospital de la Ribera, Alzira. All patients or their relatives were fully informed of the aims and scope of the research and signed an informed consent.

Sardinian cohort is described in [43].

### Peripheral blood mononuclear cells isolation

Whole blood collected in one VACUTAINER® CPT™ (BD, Franklin Lakes, NJ) containing sodium heparin as anticoagulantwas collected from each subject. Within 0.5 hours of collection, blood was processed at the collection site according to the manufacturer's instructions by centrifugation at 3000 × g at room temperature for 15 minutes. After centrifugation, the CPTs were gently inverted several times to separate plasma, mononuclear cells, and erythrocytes. We collected the white ring containing mononuclear cells. Mononuclear cells were washed twice in PBS and frozen at −80°C for subsequent RNA isolation.

### Isolation of total RNA from peripheral blood mononuclear cells

Total RNA was isolated using a mirVanamiRNA Isolation Kit (Ambion, Austin, TX) according to the manufacturer's directions. The purity and concentration of RNA were determined from OD260/280 readings byGenequant Pro Classic spectrophotometer (GE Healthcare, Little Chalfont, UK). RNA integrity was determined by capillary electrophoresis using the RNA 6000 Nano Lab-on-a-Chip kit and the Bioanalyzer 2100 (Agilent Technologies, Santa Clara, CA). Only RNA extracts with RNA integrity no. values ≥6 underwent further analysis.

### Gene expression profiling

mRNA profiling was performed using GeneChip Human Gene 1.0ST Array (Affymetrix, Santa Clara, CA, USA). This array comprises>750,000 unique 25-mer oligonucleotide features constituting over 28,000 gene-level probe sets.

Microarray experiments were conducted according to the manufacturer's instructions. Briefly, 200 ng total RNA was labeled using WT Expression Kit (Ambion). The labeling reaction was hybridized on the Human Gene Array in Hybridization Oven 640 (Affymetrix) at 45°C for 18hours. The eight arrays for each group were stained with Fluidics Station 450 using fluidics script FS450_0007 (Affymetrix) then scanned on GeneChip Scanner 3000 7G (Affymetrix). GeneChip® Command Console®software supplied by Affymetrix was used to perform gene expression analysis. All raw data regarding mRNA expression has been deposited in ArrayExpress, a publicly accessible database.

### Microarray data analysis

Data (.CEL files) were analyzed and statistically filtered using software Partek Genomic Suite 6.4 (Partek Inc., St. Louis, MO). Input files were normalized with the RMA algorithm for gene array on core metaprobesets. A 1-way ANOVA was performed with the Partek Genomics Suite across all samples. Statistically significant genes between different groups were identified using a model analysis of variance with P-value ≤0.05. The imported data were analyzed by Principal Components Analysis to determine the significant sources of variability in the data. Finally, the selected genes, specified for centenarian group, were imported into Pathway Studio v8 (Ariadne software) to classify the molecular function and biological processes represented by the mRNAs differentially expressed in the intersection between centenarians versus young and centenarians versus normal aging. Sub-networks were generated by connecting genes to their neighbors in the database (ResNet 8.0; 2010Q4 Mammal). The choice of neighbors was for expression targets. The filter used was ≥2 selected genes should be present in a sub-network. Sub-networks with the best P-value of 0.05 enrichment P-value cutoff were selected.

### Real-time PCR validation

RT-PCR was performed with random hexamers using MultiScribe™reverse transcriptase (Applied Bio-systems). First-strand cDNA synthesis was performed at 42°C for 30 minutes. The reaction was stopped by heating the mixture at 95°C for 5 minutes; tubes were stored at −20°C until further use. Pre-developed Taqman primers specific for BCl-xL (Hs00236329_m1)were purchased from Applied Biosystems. The transcript levels were detected by the 7900HT Fast Real-Time PCR System (Applied Biosystems). Each PCR reaction contains 1 μL RT product, 5 μL Taqman Gene Expression Master Mix (Applied Biosystems), and 0.5 μL probes in a final volume of 10 μL. The PCR conditions were as follows: 50°C for 2 minutes, 95°C for 10 minutes, followed by 40 cycles of 95°C for 15 seconds and 60°C for 1 minute. All PCR reactions were cycled in the linear region of amplification. Results were normalized according to RPLPO (housekeeping control, ref: 4333761F, Applied Biosystems) quantification in the same sample reaction. The threshold cycle (CT) was determined then the relative gene expression was determined as follows:

Relative amount = 2–Δ(ΔCT)

WhereΔCT =CTtarget – CThousekeeping (control) and Δ(ΔCT) = ΔCTstudied–ΔCTbaseline.

Gene level in young group was chosen as the baseline.

### Apoptosis markers

To determine cytochrome c concentration in plasma we used enzyme-linked immunosorbent assay (ELISA). Cytochrome c was quantified from a standard curve using purified Cyt c supplied in commercial ELISA kits for human Cyt c (Antibodies-online.com); samples were processed according to the manufacturer's instructions.

**Mitochondrial membrane potential**

Mitochondrial membrane potential (MMP) was measured by the lipophilic cationic probe 5,5%,6,6%-tetrachloro-1,1%,3,3%-tetraethylbenzimidazolcarbo-cyanine iodide (JC-1; Molecular Probes, Eugene, OR) using a method that has been previously applied to apoptotic cells26. The cell suspension was adjusted to a density of 0.2 × 106cells/mL and incubated with 10 μg/:mL JC-1 in complete medium in the dark at 37°C for 30 minutes. At the end of the incubation period, the cells were washed twice in phosphate buffered saline (PBS), resuspended in a total volume of 400 mL, and analyzed by flow cytometer.

### Peripheral lymphocyte function

In a separate cohort of 85 Spanish centenarians who were recruited in Madrid peripheral blood was collected and similarly processed as described above. Chemotaxis capacity of lymphocytes was performed according to a modification of Puerto et al. [34], which is itself a modification of the method described by Boyden35, basically that employs chambers with two compartments separated by a filter (Millipore) with a pore diameter of 3 μm. Aliquots of 0.3 mL of the peripheral lymphocytes suspension (106cells/mL in Hank's medium) were deposited in the upper compartment of the Boyden chambers. F-met-leu-phe (Sigma) (a positive chemotactic peptide in vitro), at 10–8 M, was placed in the lower compartment so as to determine chemotaxis. The chambers were incubated in 5% CO2 at 37°C for 3 hoursthen the filters were fixed, stained, and the chemotaxis index (CI) was determined by counting in an optical microscope (immersion objective) the total number of lymphocytes in one third of the lower face of the filters. Natural killer cell activity was assessed as follows. An enzymatic colorimetric assay (Cytotox 96™Promega; Boeringher-Ingelheim, Germany) was used to assess target cell (human tumoral K562 cells) cytolysis. Target cells were seeded in 96-well U bottom culture plates (Orange Scientific, Belgium) at 104 cells/well in 1640 RPMI without phenol red (PAA Laboratories GmbH, Austria). Effector cells (PBMCs) were added at 105 cells/well—hence effector/target ratio was 10:1. Plates were centrifuged at 250 g for 5 minutes so as to facilitate cell contacts and incubated thereafter at 37°C in a humidified atmosphere of 5% CO2 for 4 hours. Then, they were again centrifuged and LDH enzymatic activity was measured in 50 μL/well of supernatants by addition of enzyme substrate and absorbance recording at 490 nm. Three control measurements were performed: target spontaneous release; target maximum release; and effector spontaneous release.

### Measurement of oxidative stress parameters

Lipid peroxides were determined as MDA accumulation by high-performance liquid chromatography as an MDA–thiobarbituric acid adduct according to the method of Wong and associates[44]. Oxidative modification of proteins was assessed by immunoblot detection of carbonyl groups in proteins using the OxyBlot™ Protein Oxidation Detection Kit (Milllipore) following the manufacturer's instructions. In this assay, the carbonyl groups in protein side chains were derivatized to 2,4-dinitrophenylhydrazone (DNP-hydrazone) by reaction with 2,4- dinitrophenyl-hydrazine (DNPH). The DNP-derivatized protein samples were separated by polyacrylamide gel electrophoresis followed by Western blotting onto a membrane filter. The filters were incubated with primary antibody specific to the DNP moiety of the proteins followed by incubation with horseradish peroxidase-antibody conjugate directed against the primary antibody (secondary antibody: goat anti-rabbit IgG). The filters were then treated with chemilumi-nescent reagents (luminol and enhancer) and exposed to blue light-sensitive films. The procedure to quantify total protein carbonyls with the OxyBlot kit was densitometry of the oxyblot and of the Ponceau staining, followed by finding the ratio between the total density in the oxyblot and that in the Ponceau.

### Assays in mouse embryo fibroblatss

Isolation, culture, and assays with MEFs were performed as described previously [32]. Bcl-xL was ectopically expressed from retroviral vector pMIG (Addgene) as described previously [33].

### RNA analysis

For real-time quantitative RT–PCR, total RNA from cells was extracted with Trizol (Life Technologies). Reverse transcription was performed using random priming and Superscript Reverse Transcriptase (Life Technologies) according to the manufacturer's guidelines. Real-time PCR was performed using an ABI PRISM 7700 (Applied Biosystems) by using DNA Master SYBR Green I mix (Applied Bio-systems). The primer sequences used were previously described [45].

### Proliferation assay

Proliferation was measured by P-Histone3 inmuno-fluorescence. Briefly, cells were fixed with 4% paraformaldehyde for 15 min, and washed with PBS supplemented with 0.2% Triton X-100 and 1% FCS, for 5 min at 4°C. Subsequent to blocking for 1h with PBS and 1% FCS, cells were incubated with P-H3 (Chemicom) antibody for 2h. Secondary antibodies were from Jackson. Nuclear DNA was stained with a 4% PBS buffered paraformaldehyde solution con-taining 10 μg/ml 4′6-diamidino-2-phenylindole (DAPI, Sigma).

### Immunofluorescence

Cells were fixed with 4% paraformaldehyde for 15 minutesthen washed with PBS supplemented with 0.2% Triton X-100 and 1% FCS at 4°C for 5 minutes. Subsequent to blocking for 1hour with PBS and 1% FCS, cells were incubated with P-H3 (Millipore) or GFP (Abcam, Cambridge, UK) antibodies for 1hour. Secondary antibodies were procured from Jackson ImmunoResearch (West Grove, PA). Nuclear DNA was stained with a 4% PBS buffered paraformaldehyde solution containing10μg/mL 4′6-diamidino-2-phenylindole (DAPI; Sigma).

### Senescence-associated β-galactosidase activity assay

SA-β-Gal activity was measured by SA-β-Gal staining kit (Cell Signaling Technology, Beverly, MA).

### Analysis in lymphocytes from septuagenarians

For PBMC culture, they were isolated using lymphoprep following the manufacturer´s instructions (Axis shield, Oslo, Norway) from septuagenarian individuals. After collecting and washing the mononuclear cells, 2×10^6^ cells were infected with a lentivirus containing Bcl-xL (pCDH-puro-Bcl-XL, Addgene, plasmid #46972) or the empty vector as previously described [46]. Cells were maintained in RPMI high glucose media supplemented with 10% FBS, 2 mM L-glutamine and penicillin (100 U/ml) and streptomycin (100 μg/ml)at 37°C in 5% CO_2_ atmosphere,in the presence or absence of the mitogen PHA 1% (Sigma, St Louis, MO). Cell pellets were harvested for RNA analysis after 9 days in culture.

### *C. elegans* lifespan analysis

All strains were maintained at 20°C by standard methods, on solid agar NGM plates and fed E. coli OP50. The MT4770 *[ced-9(n1950)]* mutant strain was crossed with wild-type in order to retrieve a wild-type and a mutant strain with the same genetic background. For each strain, eggs were isolated from gravid adults by bleaching (NaOCl 10-50%, KOH 5M, ddH_2_0). Eggs were then placed in M9 1X medium overnight to obtain a synchronized population of L1 worms that were then plated. 150 worms were transferred onto 10 freshly seeded plates (15 worms/plate) at day 1, and changed to new plates every day. Worms were scored as dead or alive by gently tapping with an eyelash every day. Plotting of the data and statistical analysis were performed using GraphPad Prism v6.0. The Gehan-Breslow-Wilcoxon test was applied.

### Statistical analysis of validation results

Data were represented by mean ± standard deviation (SD). Comparison between groups was performed with a one-way ANOVA and two-tailed t-test. P-values <0.05 were considered statistically significant.

## SUPPLEMENTARY MATERIAL TABLE AND FIGURES


