# Supplementary material for: Human exceptional longevity: transcriptome from centenarians is distinct from septuagenarians and reveals a role of Bcl-xL in successful aging
Source: Aging (Albany NY). 2016 Oct 28;8(12):3185–201. doi: 10.18632/aging.101078 (PMC5270663; doi:10.18632/aging.101078)
Supplement: Supplementary file 1 [file aging-08-3185-s001.pdf]

## SUPPLEMENTARY MATERIAL

**Supplementary Table 1. List of the hundred processes that are more involved in exceptional longevity.** These processes are ordered by p value from the highest to the lowest.

| Order according to p value | Name | Number of genes |
|---|---|---|
| 1 | immune response | 63 |
| 2 | cell adhesion | 58 |
| 3 | MHC class I receptor activity | 9 |
| 4 | transport | 97 |
| 5 | antigen processing and presentation of peptide antigen via MHC class I | 9 |
| 6 | cellular defense response | 14 |
| 7 | response to drug | 35 |
| 8 | ion transport | 44 |
| 9 | interspecies interaction between organisms | 27 |
| 10 | signal transduction | 129 |
| 11 | cell surface receptor linked signaling pathway | 24 |
| 12 | small GTPase mediated signal transduction | 26 |
| 13 | intracellular signaling pathway | 30 |
| 14 | response to wounding | 13 |
| 15 | Presentation of endogenous peptide antigen | 5 |
| 16 | response to hypoxia | 21 |
| 17 | apoptosis | 40 |
| 18 | protein transport | 36 |
| 19 | T cell activation | 9 |
| 20 | integral to plasma membrane | 79 |
| 21 | response to lipopolysaccharide | 17 |
| 22 | actin binding | 30 |
| 23 | myoblast fusion | 5 |
| 24 | GTPase activator activity | 21 |
| 25 | regulation of blood volume by renal aldosterone | 3 |
| 26 | nucleotide binding | 113 |
| 27 | cytoskeleton | 60 |
| 28 | post-Golgi vesicle-mediated transport | 8 |
| 29 | positive regulation of T cell mediated cytotoxicity | 5 |
| 30 | membrane raft | 18 |
| 31 | GTP binding | 34 |
| 32 | Oncogenes | 27 |
| 33 | cellular response to growth factor stimulus | 7 |
| 34 | cell surface | 28 |

| | | |
|---|---|---|
| 35 | embryonic digit morphogenesis | **7** |
| 36 | calmodulin binding | 16 |
| 37 | metabolic process | 43 |
| 38 | skeletal system development | 14 |
| 39 | positive regulation of transcription from RNA polymerase II promoter | 27 |
| 40 | NK Cell Activation | 50 |
| 41 | vesicle-mediated transport | 18 |
| 42 | integrin binding | 11 |
| 43 | protein amino acid phosphorylation | 38 |
| 44 | Golgi apparatus | 61 |
| 45 | negative regulation of epithelial cell migration | 3 |
| 46 | cytoskeletal anchoring at nuclear membrane | 3 |
| 47 | endoplasmic reticulum | 67 |
| 48 | hydrolase activity | 85 |
| 49 | cellular component movement | 12 |
| 50 | induction of apoptosis by extracellular signals | 11 |
| 51 | regulation of transcription | 81 |
| 52 | wound healing | 10 |
| 53 | microtubule | 26 |
| 54 | negative regulation of T cell activation | 4 |
| 55 | response to stress | 18 |
| 56 | regulation of G-protein coupled receptor protein signaling pathway | 7 |
| 57 | T cell receptor signaling pathway | 6 |
| 58 | peptide antigen binding | 6 |
| 59 | NAD+ ADP-ribosyltransferase activity | 6 |
| 60 | endosome | 26 |
| 61 | hematopoietin-interferon-class (D200-domain) cytokine receptor signal transducer activity | 3 |
| 62 | chemotaxis | 13 |
| 63 | SH3-SH2 adaptor activity | 8 |
| 64 | positive regulation of triglyceride biosynthetic process | 3 |
| 65 | thrombin receptor signaling pathway | 3 |
| 66 | integral to membrane of membrane fraction | 6 |
| 67 | activation of pro-apoptotic gene products | 5 |
| 68 | regulation of smoothened signaling pathway | 4 |
| 69 | mitochondrion | 89 |
| 70 | positive regulation of T cell proliferation | 6 |
| 71 | response to organic cyclic substance | 16 |
| 72 | antigen processing and presentation | 10 |
| 73 | platelet dense granule membrane | 3 |
| 74 | SUN-KASH complex | 3 |

| | | |
|---|---|---|
| 75 | eukaryotic cell surface binding | **5** |
| 76 | myelination | 7 |
| 77 | C21-steroid hormone biosynthetic process | 3 |
| 78 | regulation of ARF GTPase activity | 6 |
| 79 | MHC class I protein complex | 9 |
| 80 | ruffle | 10 |
| 81 | cytokine-mediated signaling pathway | 9 |
| 82 | evasion of host defenses by virus | 2 |
| 83 | negative regulation of calcium-mediated signaling | 2 |
| 84 | interleukin-2 secretion | 2 |
| 85 | cortisol biosynthetic process | 2 |
| 86 | cellular response to antibiotic | 2 |
| 87 | positive regulation of plasma membrane long-chain fatty acid transport | 2 |
| 88 | PAS domain | 5 |
| 89 | cell proliferation | 24 |
| 90 | cellular response to insulin stimulus | 8 |
| 91 | Transcytosis | 4 |
| 92 | CCR1 -> STAT signaling | 5 |
| 93 | metal ion binding | 150 |
| 94 | actin cytoskeleton reorganization | 5 |
| 95 | NAD or NADH binding | 9 |
| 96 | protein ubiquitination | 12 |
| 97 | protein kinase activity | 34 |
| 98 | lipopolysaccharide-mediated signaling pathway | 4 |
| 99 | negative regulation of T cell receptor signaling pathway | 3 |
| 100 | chemokine receptor activity | 5 |

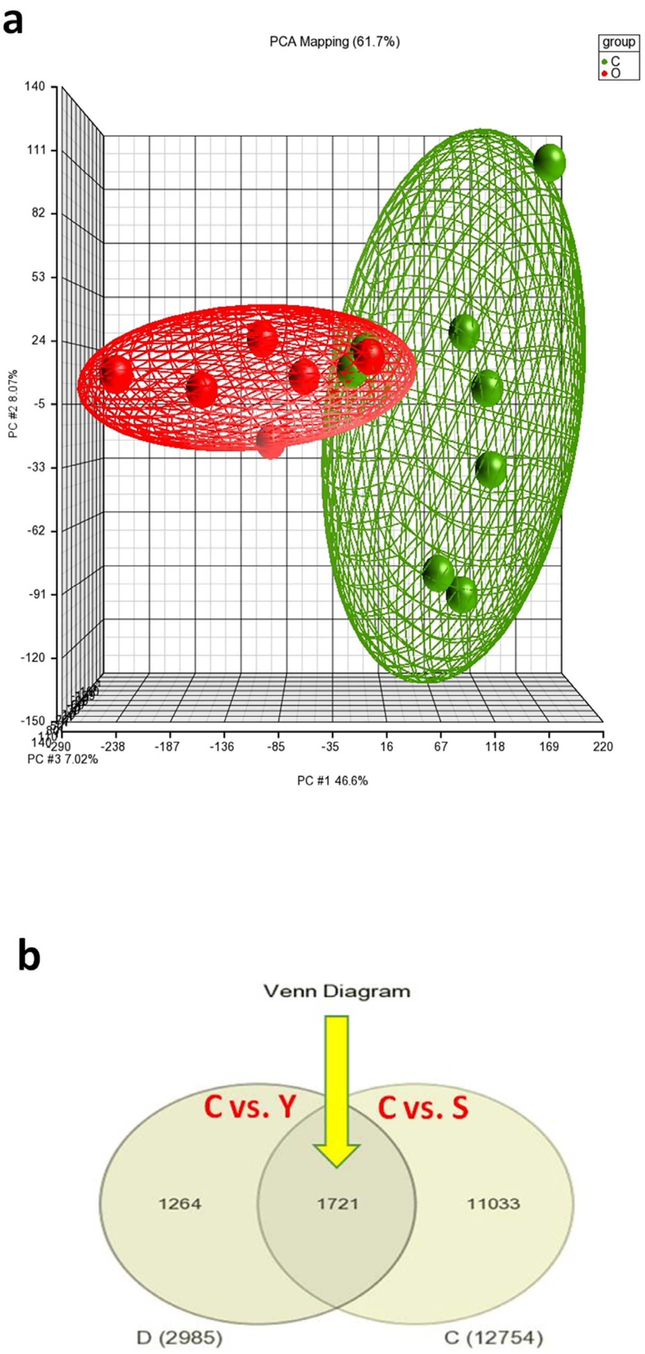


**Supplementary Figure 1.** (**a**) Principal component analysis (PCA) of mRNA profiles in mononuclear cells from septuagenarians and centenarians. Each sample was assayed by GeneChip Human Gene 1.0ST Array. The ellipsoids (red, septuagenarians; n=6; green, centenarians; n=8) show distinct directionalities. The x–, y–, and z-axes correspond to principal component (PC)1, PC2, and PC3, respectively. (**b**) Venn diagram displaying no. mRNAs commonly regulated in the different age groups. No. mRNAs that are significantly modified when comparing young (n=8), septuagenarians (n=8), and centenarians (n=8). Data (.CEL files) were analyzed and statistically filtered using Partek Genomic Suite 6.4software. Statistically significant mRNAs were filtered by applying P-value <0.05 and fold change ≥ |1.8|.

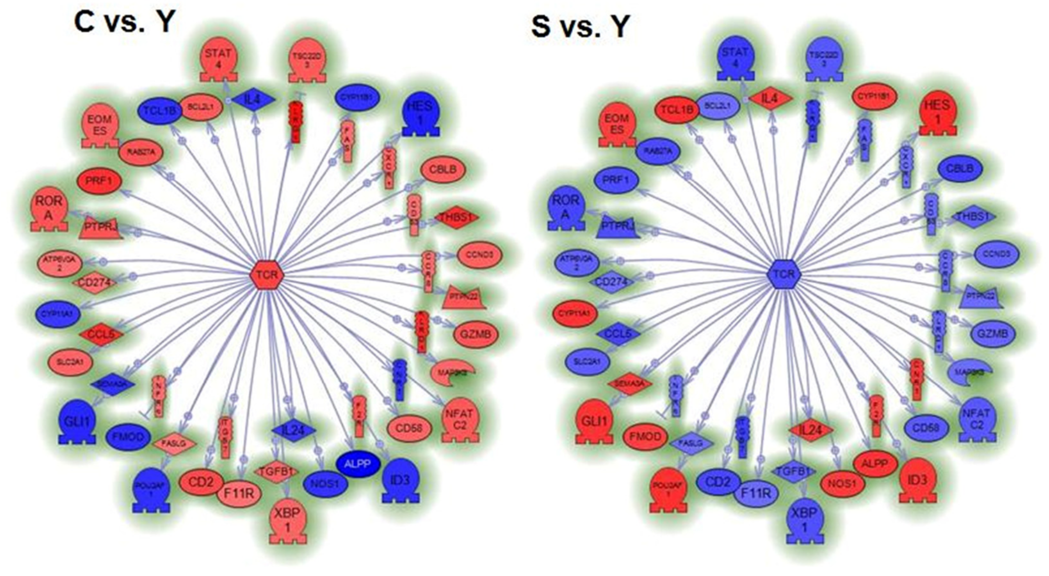


**Supplementary Figure 2. Sub-network analysis of genes specifically regulated by centenarians or septuagenarians versus young subjects indicates the relevance of T cell receptor (TCR).** Sub-network of genes regulated by TCR in mononuclear cells obtained from centenarians (**a**) or septuagenarians (**b**) versus young subjects. Sub-networks are generated by connecting genes to their neighbors in the database (ResNet 8.0; 2010Q4 Mammal). Sub-networks with enrichment P-value <0.05 cutoff were selected.

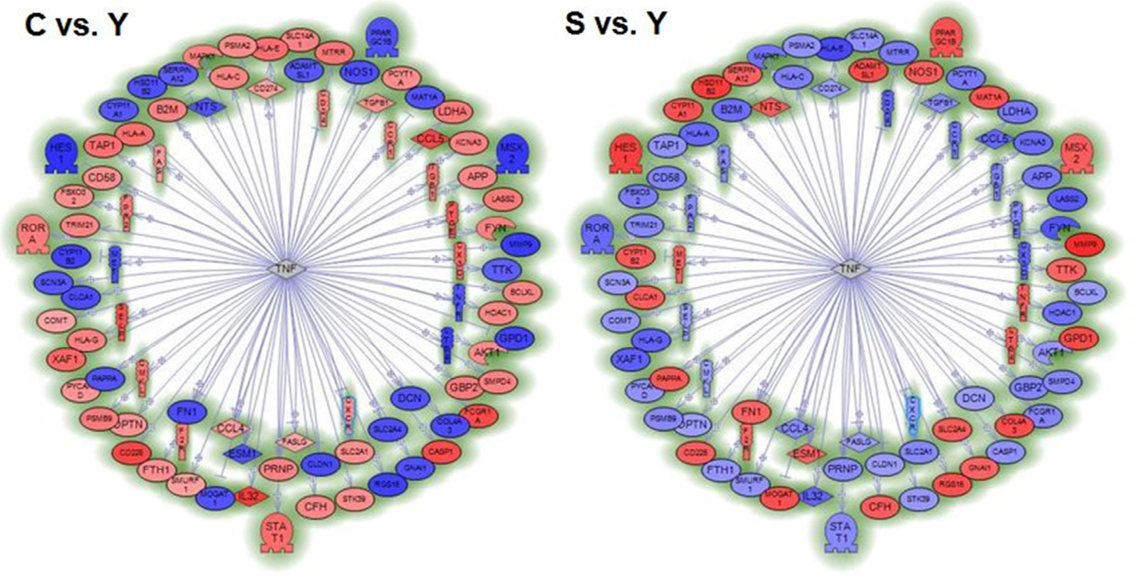


**Supplementary Figure 3. Sub-network analysis of genes specifically regulated by centenarians or septuagenarians versus young subjects indicates the relevance of tumor necrosis factor (TNF).** Sub-network of genes regulated by TNF in mononuclear cells obtained from centenarians (**a**) or septuagenarians (**b**) versus young subjects. Sub-networks are generated by connecting genes to their neighbors in the database (ResNet 8.0; 2010Q4 Mammal). Sub-networks with enrichment P-value <0.05 cutoff were selected.

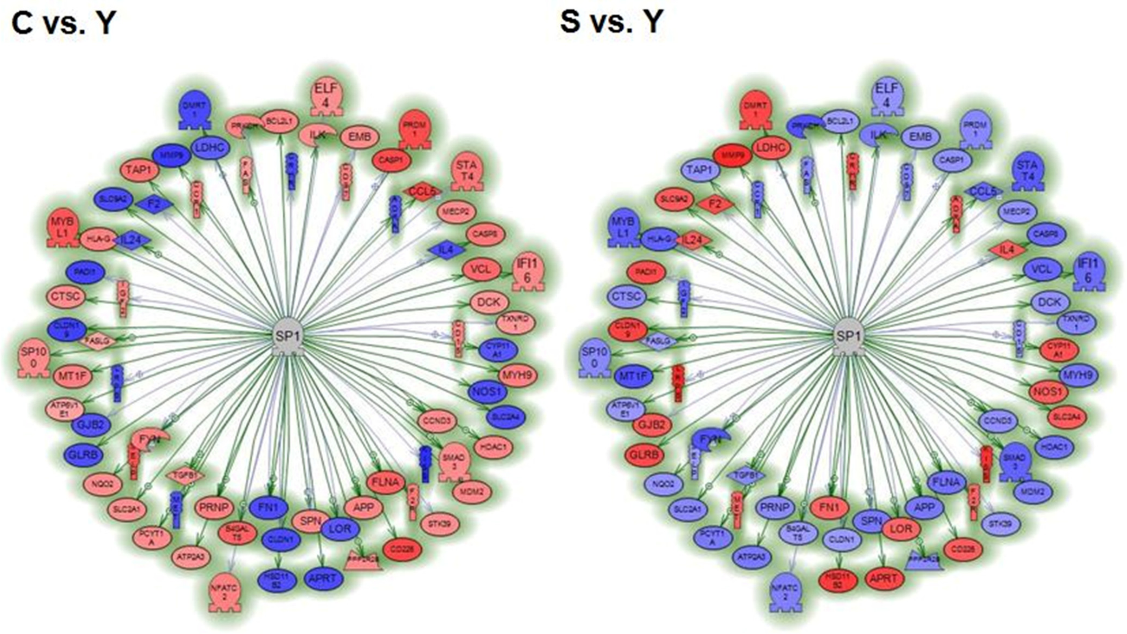


**Supplementary Figure 4. Sub-network analysis of genes specifically regulated by centenarians or septuagenarians versus young subjects indicates the relevance of SP1.** Sub-network of genes regulated by SP1 transcription factor in mononuclear cells obtained from centenarians (a) or septuagenarians (b) versus young subjects. Sub-networks are generated by connecting genes to their neighbors in the database (ResNet 8.0; 2010Q4 Mammal). Sub-networks with enrichment P-value <0.05 cutoff were selected.

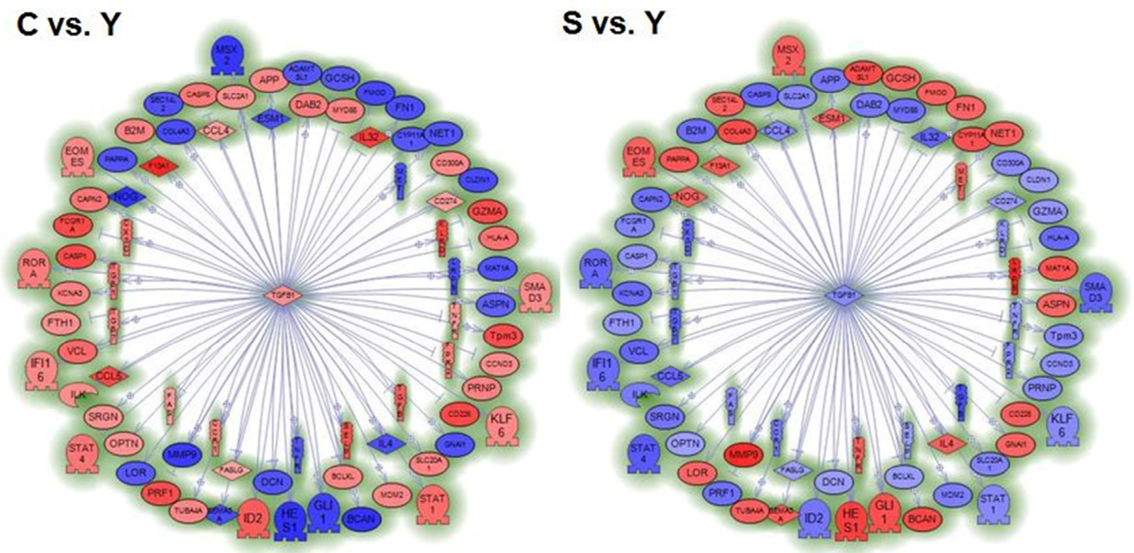


**Supplementary Figure 5. Sub-network analysis of genes specifically regulated by centenarians or septuagenarians versus young subjects indicates the relevance of TGFB1.** Sub-network of genes regulated by transforming growth factor(TGF)-β1 (TGFB1) in mononuclear cells obtained from centenarians (**a**) or septuagenarians (**b**) versus young subjects. Sub-networks are generated by connecting genes to their neighbors in the database (ResNet 8.0; 2010Q4 Mammal). Sub-networks with enrichment P-value <0.05 cutoff were selected.

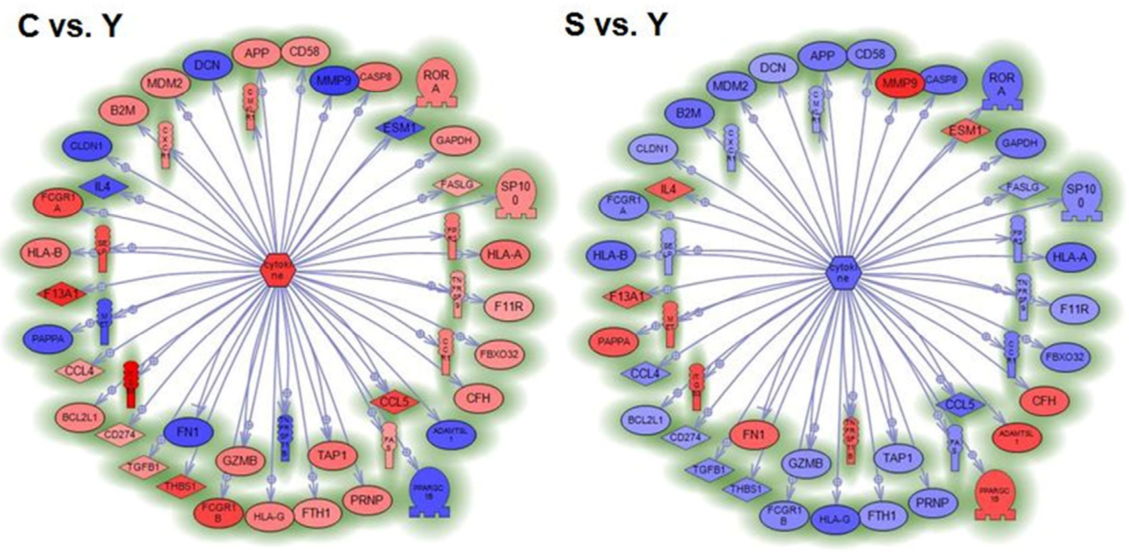


**Supplementary Figure 6. Sub-network analysis of genes specifically regulated by centenarians or septuagenarians versus young subjects indicates the relevance of cytokine IL-32.** Sub-network of genes regulated by IL-32in mononuclear cells obtained from centenarians (a) or septuagenarians (b) versus young subjects. Sub-networks are generated by connecting genes to their neighbors in the database (ResNet 8.0; 2010Q4 Mammal). Sub-networks with enrichment P-value <0.05 cutoff were selected.

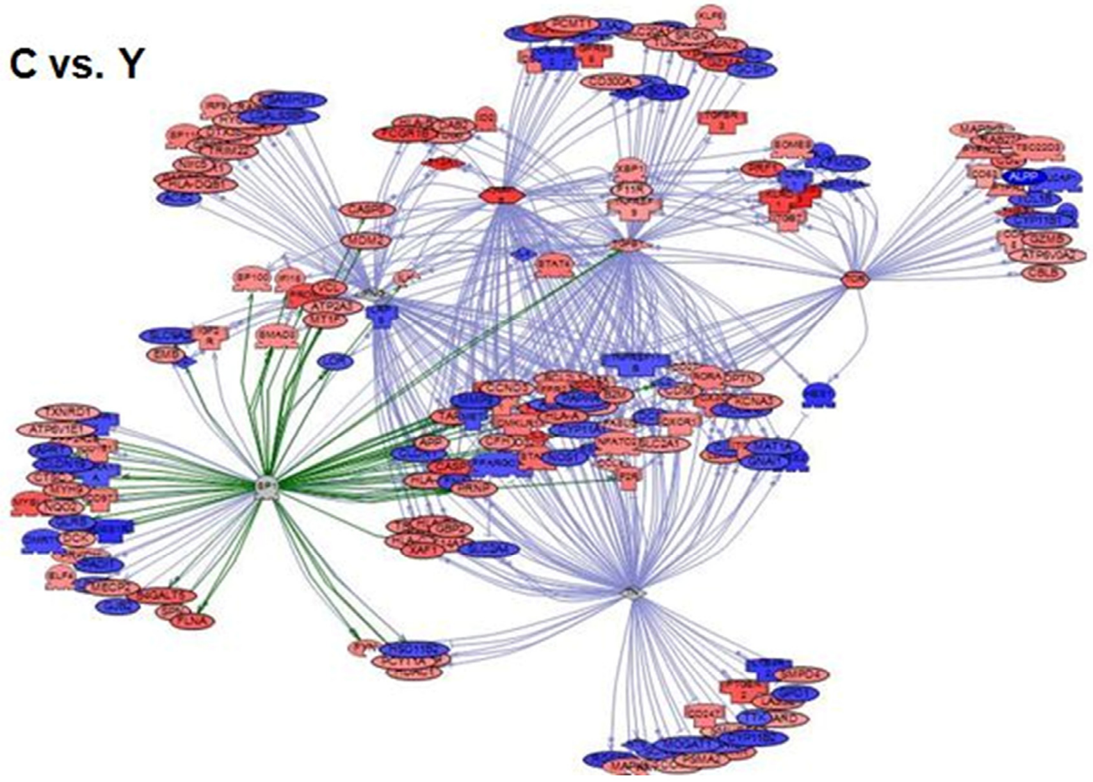


**Supplementary Figure 7. All Sub-network analysis of genes specifically regulated by centenarians or septuagenarians versus young subjects.** Sub-networks are generated by connecting genes to their neighbors in the database (ResNet 8.0; 2010Q4 Mammal). Sub-networks with enrichment P-value <0.05 cutoff were selected.
